# CT and MRI Features of Hairy Polyps in Neonates and Infants: A Retrospective Study of 14 Patients

**DOI:** 10.3390/diagnostics13071328

**Published:** 2023-04-03

**Authors:** Shuangfeng Yang, Hang Li, Jun Gao, Zhonglong Han, Xiaolu Tang, Rongchang Wu, Huiying Kang, Fengzhen Zhang, Jiatong Xu, Yun Peng

**Affiliations:** 1 Department of Radiology, Beijing Children’s Hospital, Capital Medical University, National Center for Children’s Health, Beijing 100045, China; 2 Department of Otolaryngology, Beijing Children’s Hospital, Capital Medical University, National Center for Children’s Health, Beijing 100045, China; 3 Department of Pathology, Beijing Children’s Hospital, Capital Medical University, National Center for Children’s Health, Beijing 100045, China

**Keywords:** hairy polyps, CT, MRI, neonate, infant

## Abstract

Background: The typical imaging findings of hairy polyps have been described mostly in case reports. This study was conducted to describe the CT and MRI features of hairy polyps and their common associated abnormalities. Methods: Medical records of 14 patients with pathological diagnosis of hairy polyps were collected for this study. For each patient, the medical records, including demographics, clinical manifestations, and imaging findings were reviewed. Results: The female-to-male ratio was 3.7:1. The age at first episode varied from birth to 2.7 years. The masses were derived from the back side of the soft palate in seven (50.0%) cases, from the lateral pharyngeal wall in four (28.6%) cases, from the soft palate in one (7.1%) case, from the nasal vestibule in one (7.1%) case, and from the parapharyngeal space in one (7.1%) case. A total of 11 (78.6%) cases presented with pedicled masses containing fat and a central core of soft tissue, there were 3 (21.4%) cases whose imaging findings were atypical, and there were 6 (42.9%) patients who had other pathologies. Conclusions: Hairy polyps typically presented as pedicled masses containing fat and a central core of soft tissue, but sometimes their imaging findings can be atypical and they can be associated with other congenital abnormalities. CT and MRI are reliable methods for the diagnosis of hairy polyps and their associated abnormalities.

## 1. Introduction

Hairy polyps are rare mature bi-germinal masses and are usually found in the oronasopharyngeal region [1]. Histopathologically, hairy polyps are composed of mesodermal and ectodermal derivatives, which are foreign to the nasopharynx, such as fibroadipose tissue, muscle, or cartilage (mesoderm), as well as mature stratified squamous epithelium with skin appendages, including eccrine sweat glands, sebaceous glands, and hair follicles (ectoderm). In the literature, the terms dermoids, hairy polyps, and choristoma are often used interchangeably [2], and they have been described as the most primitive form of teratoma [3]. Currently, the pathogenesis of hairy polyps is not clear, but there are several possible opinions. On the one hand, hairy polyps are classified as choristomas that may be derived from normal multifunctional tissue, and they are found in the form of irregular masses in locations different from where they are normally found. On the other hand, hairy polyps are considered developmental malformations due to abnormal embryogenesis of the first and second branchial arches [4].

Hairy polyps are nonmalignant lesions with limited growth potential and mostly occur in newborns, infants, and young children, with a higher prevalence in females [5]. The incidence of hairy polyps is reportedly less than 1:40,000 live births [5]. Hairy polyps are unusual causes of respiratory distress in newborns and infants; however, despite their rarity, they are considered the most common congenital nasopharyngeal masses [5]. Clinical symptoms depend on the location and size of the masses. They typically present with respiratory obstruction or feeding difficulties at or shortly after birth [6]. They are usually isolated events but have been described in association with other congenital abnormalities. The clinical diagnosis situation of this mass varies. Most cases are diagnosed with clinical examination or endoscopy, with a polypoid mass being seen [7]. However, the mass can be obscured by the endotracheal tube, and thus escape clinical recognition during oral examination if it is small [8]. Misdiagnosis and missed diagnosis of hairy polyps often occur. Imaging examinations are useful for delineating the size, origin, and extent of the masses and are essential for the determination of intracranial extension and especially evaluating associated comorbidities that can be easily missed at an initial visit. At present, there are limited reports on hairy polyps, and most of them are case reports on clinical and pathological findings. Reports on imaging findings are extremely rare, and the number of cases is very small. In this study, we analyzed a larger cohort of hairy polyps to evaluate diagnostic features on cross sectional imaging.

## 2. Material and Methods

### 2.1. Study Cohort

Twenty-four cases of hairy polyps confirmed by pathology between January 2013 and March 2020 were retrospectively reviewed; however, ten cases without preoperative CT or MRI scans were ruled out. This work was approved by the local ethics board. All 14 patients with medical records, including demographics, clinical manifestations, CT, and/or MRI findings, were reviewed. A total of 3 patients had undergone MRI, 6 patients had undergone CT, and 5 of them had received both MRI and CT. Out of the 14 patients in this study, 3 patients received contrast. One patient received contrast-enhanced CT scans. One patient received contrast-enhanced MRI scans. One patient received both contrast-enhanced CT and MRI scans. The location, origin, morphology, size, density/signal characteristics, and contrast enhancement pattern of the masses were analyzed on the CT and MRI images.

### 2.2. MRI Scans

MRI scans were performed with a 3.0T MR scanner (Achieva, Philips Medical Systems, Best, The Netherlands) with a 16-channel phased-array receiver coil in the referral area. All cases received transverse T1-weighted or T2-weighted, sagittal, or coronal T2-weighted scan, and transverse, sagittal, and coronal T2-weighted spectral presaturation and inversion recovery (T2W-SPIR). Gadolinium-enhanced T1W-SPIR was not standard and was used in two patients. Diffusion-weighted imaging (DWI) was used in five cases. A gradient strength of b = 1000 s/mm^2^ was used for DWI. Additional sequences were acquired according to the patient’s condition.

### 2.3. CT Scans

CT scans were performed using a 256-row CT scanner (Revolution CT, GE Healthcare, Chicago, IL, USA) from the base of the skull to the level of the thoracic inlet. The scan was performed once before and once after the injection of the contrast medium. Iodixanol-enhanced CT scans were not standard and were used in two patients. The tube voltages of the plain scan, arterial phase, and venous phase were 120 KVp, 80 KVp, and 120 KVp, respectively. The tube current was set by automatic tube current modulation.

## 3. Results

Clinical and imaging data of 14 patients are shown in Table 1.

### 3.1. Clinical Findings

There were 11 females and 3 males with a female-to-male ratio of 3.7:1. There were 11 neonates. The age at first episode varied from birth to 2.7 years with a median age of 1 day. The clinical manifestations included dyspnea (42.9%), polypnea (35.7%), snoring (21.4%), nasal obstruction (21.4%), mouth breathing (21.4%), perioral cyanosis (21.4%), choking on milk (21.4%), hearing impairment (14.3%), difficulty feeding (7.1%), otopyorrhea (7.1%), inspiratory stridor (7.1%), gurgling with sputum (7.1%), and heart rate reduction (14.3%). Three patients underwent cardiopulmonary resuscitation (CPR) due to mass-induced asphyxia. One patient was asymptomatic, and the pharyngeal mass was detected by accident when being treated for a cold.

Of the 14 patients, the mass occurred on the left side in 9 (64.3%) patients, on the right side in 4 (28.6%) patients, and on the midline in 1 (7.1%) patient. Intraoperative findings showed that the lesions were derived from the back side of the soft palate in seven (50.0%) cases; from the lateral pharyngeal wall in four (28.6%) cases, including the pharyngeal opening of auditory tube, the eustachian tube, the pharyngeal recess, and the inferior side of the pharyngeal opening of the auditory tube; from the soft palate in one (7.1%) case; from the nasal vestibule in one (7.1%) case; and from the parapharyngeal space extending to external the auditory canal and middle ear in one (7.1%) case. The main body of the mass was located in the pharyngeal region in 11 (78.6%) cases, in the nasal cavity and nasopharynx in 1 (7.1%) case, in the nasal vestibule in 1 (7.1%) case, and in the parapharyngeal space, the external auditory canal, and the tympanum in 1 (7.1%) case. There was one case where the lesion was stretched to the oral cavity during a severe cough.

### 3.2. Imaging Findings

**Figure 1:**
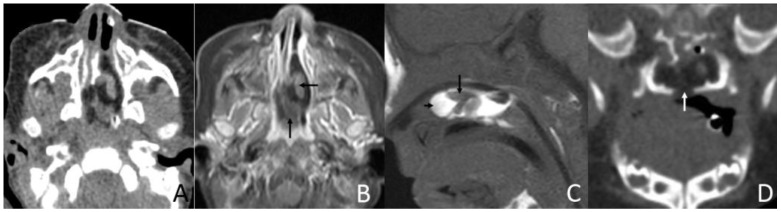
CT and MRI of a 2-month and 17-day girl presenting shortly after birth with dyspnea and inspiratory stridor. (**A**) Axial CT, displayed in the soft-tissue window, shows the soft palate mass protruding to the left nasal cavity and blocking the bilateral choanae to be of predominantly low attenuation, containing an irregular moderate-attenuation stalk. (**B**) Axial, postcontrast, T1W-SPIR MRI shows that the lesion’s stalk is slightly enhanced (arrows), but the rest of the lesion is not enhanced. (**C**) Sagittal T1-weighted MRI shows an irregular mass in the soft palate and left nasal cavity that mainly has a high-signal (short arrow) with an irregular low-signal central stalk (long arrow). (**D**) Coronal CT, displayed in the bone window, shows the soft palate mass associated with the cleft palate (arrow).

**Figure 2:**
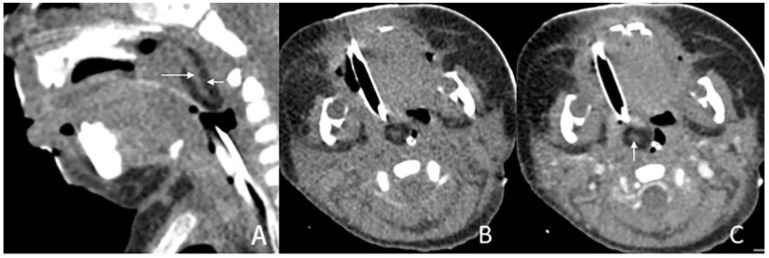
CT of a 1-month and 16-day girl presenting with throat congestion 29 days after birth. (**A**,**B**) Sagittal and axial CT, displayed in the soft-tissue window, shows a sausage-like fat density mass (short arrow) with a soft tissue density core (long arrow) originating from the left lateral pharyngeal wall. (**C**) Axial, postcontrast CT, displayed in the soft-tissue window, shows that the lesion’s stalk is slightly enhanced (arrow), but the rest of the lesion is not enhanced.

The size of masses varied from 0.6 to 4.3 cm. There were 11 (78.6%) cases with the largest diameter of the mass greater than 2 cm. The shapes of the masses were varied. Eleven (78.6%) cases presented with typical pedicled masses containing fat and a central core of soft tissue, including sausage-like masses in five (35.7%) cases, tongue-like masses in four (28.6%) cases, and pear-like masses in two (14.3%) cases. Of the eight patients who underwent MRI scans, there were five (62.5%) patients whose masses had high signal intensity on T1-weighted and T2-weighted sequences with a low-signal central core whose signal intensities were similar to those of muscles. The high signal intensities on all sequences were similar to the fat signals and attenuated by fat suppression. The central core demonstrated mild enhancement following intravenous gadolinium administration, while the surrounding components of the fat signals demonstrated no enhancement in one case (Figure 1). There was no reduced diffusion of the five patients who underwent DWI. Via CT, there were 10 (83.3%) cases whose masses appeared as well-circumscribed fat attenuation masses surrounding a central core of soft tissue. The central core was mildly enhanced following intravenous iodixanol administration, but the fat components were not enhanced in one case (Figure 2). There were five patients who received both MRI and CT.

**Figure 3:**
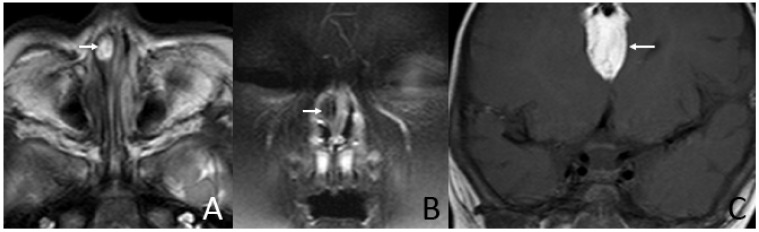
MRI of a 6-month-old and 18-day-old girl presenting shortly after birth with nasal obstruction. (**A**) Axial T2-weighted MRI shows a sausage-like mass (arrow) in the right nasal vestibule and nasal limen that is mainly high-signal with linear low-signal inside. (**B**) Coronal T2W-SPIR MRI shows the hyperintensity component of the mass suppressed (arrow). (**C**) Coronal T1-weighted MRI shows an irregular mass (arrow) containing fat in the intracranial midline that mainly has a high-signal with irregular linear low-signal inside.

**Figure 4:**
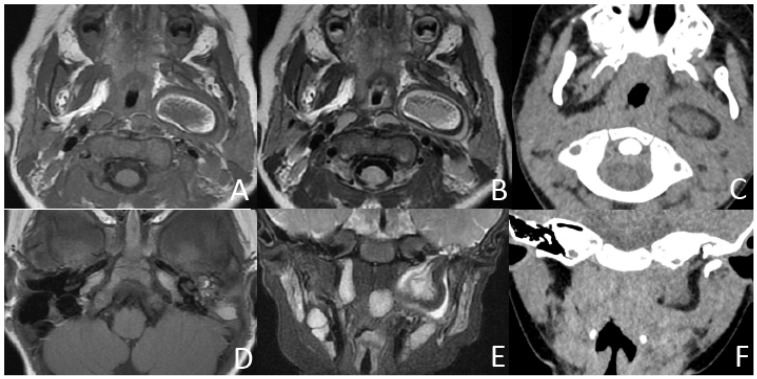
MRI (conducted at 11 months) and CT (conducted at 2 years and 7 months) of a girl who failed newborn hearing screening of the left ear, presenting with left otopyorrhea from 2 years old. (**A**,**B**) Axial T1-weighted, T2-weighted MRI show a multilayered circumferential mass in the left parapharyngeal space that is soft tissue, lipid, fibrous capsule, and soft tissue intense from inside to outside. (**C**) Axial CT shows a 3-layered circumferential mass that is soft tissue dense, fat dense, and soft tissue dense from inside to outside. (**D**) Axial T1-weighted MRI shows an irregular hyperintense and isointense mass in the left middle ear. (**E**,**F**) Coronal T2W-SPIR MRI and CT show the pear-like multilayered circumferential mass in the left multilayered circumferential mass with the pedicle extending to the left middle ear and the bone defect of the inferior wall of the external auditory canal and mastoid process.

**Figure 5:**
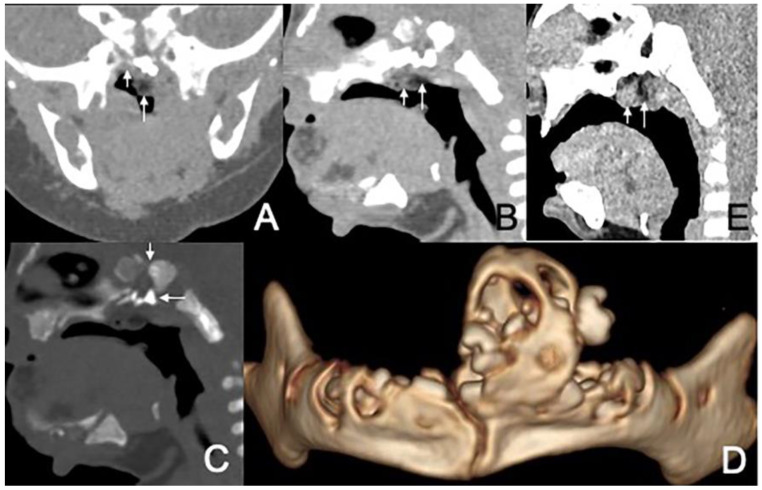
CT of a 1-month and 6-day girl presenting shortly after birth due to being unable to suck and having a mandibular mass. (**A**,**B**) Coronal and sagittal CT, displayed in the soft-tissue window, show the irregular fat (short arrow) and soft tissue (long arrow) density mass originating from the back side of the left soft palate. (**C**) Sagittal CT, displayed in the bone window, shows that there were multiple irregular dense shadows (long arrow) above the mass that were not seen, were excised during the transnasopharyngeal plasma surgery, and were probably ectopic teeth. In addition, the central bone defect of the sphenoid body was considered the remnant of craniopharyngeal canal development (short arrow). Intraoperatively, the nasopharyngeal apex wall was found intact, and no clear fluid outflow was observed. This patient was associated with cleft palate (not shown). (**D**) 3D reconstruction CT showing the associated ameloblastic fibro-odontoma of the mandible. (**E**) Sagittal CT after 3 years, displayed in a soft window, shows that the volume of the mass was increased slightly.

In addition, three (21.4%) cases presented with irregular shapes the MRI or CT findings that were atypical. One case presented with a sausage-like mass in the right nasal vestibule and nasal limen that was mainly high-signal with multiple filament-like low signals inside on T1-weighted and T2-weighted MRI (Figure 3). One case presented with a multilayered circumferential mass in the left parapharyngeal space that was soft tissue, lipid, fibrous capsule, and soft tissue intense from inside to outside, and the mass extended to the left external auditory canal and middle ear (Figure 4). One case presented with irregular fat and soft tissue density mass originating from the back side of the left soft palate (Figure 5).

### 3.3. Associated Malformation

**Figure 6:**
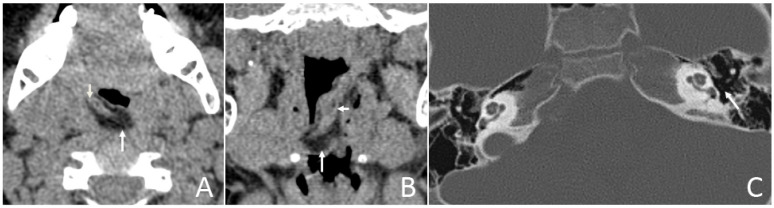
CT of a 2-year and 11-month boy presenting with snoring and mouth breathing for 3 months and hearing impairment found during physical examination. (**A**,**B**) Axial and coronal CT displayed in the soft-tissue window show the fat density mass (long arrow) originating from the back side of the left soft palate with a soft tissue density core (short arrow). (**C**) Axial CT, displayed in the bone window, shows the absence of the left stapes head and part of the stapes arch (arrow).

Six (42.9%) patients had other abnormalities. Five of them were incidental findings within the imaging scope of their investigations and one was in the other area. One infant had ossicular chain disruption showing the absence of the left stapes head and part of stapes arch (Figure 6), one had a cystic lesion of liver by abdomen ultrasound, one had frontal midline lipoma (Figure 3), one had a cleft palate (Figure 1) and pectus excavatum, one had a right first branchial fistula, and one had a cleft palate and ameloblastic fibro-odontoma of the mandible as well as highly suspicious ectopic teeth and the remnant of craniopharyngeal canal development (Figure 5).

None of the patients were associated with syndromic disorders.

### 3.4. Treatment

Complete resection was performed in all 14 cases, which were followed up for more than 1 year without recurrence.

### 3.5. Radiation Dose

The volumetric CT dose index (CTDIvol) was 2.98 ± 2.21 mGy, and the dose–length product (DLP) value was 62.50 ± 57.38 mGy-cm.

## 4. Discussion

Hairy polyps are rare congenital benign lesions, which were first described in 1784 [9]. They mostly occur in newborns, infants, and young children but are occasionally found in adulthood [10,11]. Hairy polyps can even be found and diagnosed in fetuses [12]. In the current study, neonates constituted almost 78.6% of the cases, which is a significant deviation from earlier studies that suggested that neonates constituted approximately 37% of the cases [5]. Among the neonates included, 85.7% of the lesions presented in infancy, which is consistent with the 73% rate reported by Dutta et al. [5]. Most studies have stated the female: male ratio to be 6:1 [13]. However, we found it to be 3.7:1, which is consistent with the ratio of 3.5:1 reported in the review by Dutta et al. [5]. The female preponderance of hairy polyps remains unexplained.

Hairy polyps can occur anywhere in the body [8]. Pharyngeal hairy polyps most commonly originate from the lateral nasopharyngeal wall or the superior aspect of the soft palate [14] but can also originate from the tonsils, tongue, hard palates, eustachian tube, and middle ear [6]. The nasopharynx accounts for approximately 78.6% of the hairy polyps in our study, while a prior study demonstrated that almost 60% are found in the nasopharynx, most commonly from the lateral nasopharyngeal wall or the eustachian tube [5]. Modern estimates suggest that 20% to 45% originate within the eustachian tube or middle ear [5], and we found it to be 21.4%, which is comparable to earlier reports. The lesion was derived from the nasal vestibule in one case in this study, which is a very rare location [15]. There was one case where the lesion was derived from the soft palate, which is not an uncommon location according to earlier reports. For hairy polyps with a longer pedicle, the location of their main body may vary with crying and swallowing movements of the infants. In this study, there was one case where the lesion was stretched to the oral cavity during a severe cough. The left side has been found to be 6.5 times more commonly involved, irrespective of the site of origin [5]. However, in this study, they were 2.5 times more likely to occur on the left side. The left-sided predilection remains unexplained.

Clinical symptoms depend on the location and size of the mass. In this paper, the size of masses varied from 0.6 to 4.3 cm, which is comparable to a previous study reporting the size of lesions ranging from 0.5 to 6 cm [9]. They typically present with respiratory obstruction or feeding difficulties [6]. The obstruction can be dramatic, causing asphyxia at or shortly after birth. Symptoms occurred shortly after birth in 57.1% of the cases. Three (21.4%) patients with large nasopharyngeal mass underwent cardiopulmonary resuscitation (CPR) due to tumor-induced asphyxia. In addition, the obstruction can be subtle from a small pedunculated polyp, which may not be found until later in life [16]. There was one asymptomatic case in the present study, and the pharyngeal mass was detected by accident during treatment for a cold. Other symptoms include stridor, shortness of breath, hemoptysis, nostril initiation, vomiting, slower weight gain than children of the same age, and unilateral eustachian tube dysfunction. If the mass derives from the middle ear and external auditory canal, hearing loss, otorrhea, and otitis media may occur [17]. In addition to the common symptoms, there was one case presenting with hearing impairment and otopyorrhea where the lesion was derived from the parapharyngeal space and external auditory canal and extended into the tympanum in the study. In addition, it is worth noting that one patient also presented with hearing impairment because of associated ossicular chain disruption.

Hairy polyps are not definitively associated with any syndromic disorder but have been reported to be associated with other congenital abnormalities such as cleft palate, uvula dysplasia, auricular deformities [18] such as microtia and low-set ears, ankyloglossia, facial hemihypertrophy, hypospadias, left carotid artery atresia, osteopetrosis [19], bifurcation of the tongue, and branchial arch anomalies [20,21]. Of particular importance is that they have been described to occur more frequently in patients with first or second branchial arch anomalies [22]. In rare cases, hairy polyps have been noted to occur with hypothalamic neuronal hamartoma [12] and polydactyly [16]. Neurological complications may occur secondary to vascular compression ischemia. In this paper, except for cleft palate and the first branchial fistula, other associated abnormalities, such as ossicular chain disruption, ameloblastic fibro-odontoma of the mandible, frontal midline lipoma, pectus excavatum, highly suspicious ectopic teeth, and remnants of craniopharyngeal canal development, have not been reported before. The associated abnormalities may be misdiagnosed at an initial visit if the imaging examination was not performed.

In the case of tracheal intubation, hairy polyps might be easily overlooked, as they are mobile and soft masses. One case of pharyngeal hairy polyp was obscured by oral examination and endotracheal intubation, but it was diagnosed by MRI [8]. Imaging is useful for delineating the size, origin, and extent of the mass, diagnosing and determining intracranial extension, and evaluating associated comorbidities, particularly in cases that are otherwise obscured by the presence of an endotracheal tube [17]. In particular, CT has an advantage in displaying the bony structure within the scanning range, such as showing the integrity of the skull base, external auditory canal, middle ear cavity, and the carotid artery tube, and in finding associated malformations such as the cleft palate and ossicular malformation. MRI was superior to CT in determining the components and scopes of the lesion as well as the relationship with the blood vessels and its surrounding soft tissue. However, MRI is limited when the lesion is mobile because of its motion or respiratory artifacts. Ultrasound (US) is quick and easy to perform, and it is useful for first-line investigation of neck masses in neonates and infants. US can demonstrate the size and location of the hairy polyps, which typically showed as defined, ovoid, and mobile masses [18].

Upon MRI, most of the masses were found to have heterogeneous high signal intensity on T1-weighted and T2-weighted sequences with a low-signal central core whose signal intensities were similar to those of muscles. The high signal intensities on all sequences were similar to the fat signals and attenuated by fat suppression. The central core demonstrated mild enhancement following intravenous gadolinium administration, while the surrounding components of the fat signals demonstrated no enhancement. The central core helps us trace the origin of the mass, which is important for the choice of surgical method. Characteristic imaging features on CT include well-circumscribed masses containing fat and linear soft tissue–density components centrally that correspond to the fibrovascular stalk [2]. The central core was mildly enhanced, as expected for fibrous tissue, but the fat components were not [23]. These findings corresponded well with the MRI and CT features previously described [24,25]. Intracranial or intraspinal extension was not found in any of the lesions in the present study, which was consistent with existing reports.

However, there were three cases whose imaging findings were atypical. All masses contain fat and soft tissue, but their distribution and proportions vary. In rare cases, the mass has a thick capsule.

The high fat content of hairy polyps demonstrated on CT and MRI scans helps to exclude some vascular and neurologic lesions, such as neuroblastoma, hemangioma, encephalomeningocele [26], and embryonic cysts of thymic, lingual, or thyroglossal origin [8]. This narrowed our differential diagnosis of a neonatal pharyngeal mass to hamartomas, teratomas, dermoids, and cholesteatoma, especially those arising in the middle ear [18]. Compared with hairy polyps, pharyngeal hamartomas tend to be more heterogeneous and are more commonly found in adults [17]. Teratomas appear as single or multiple masses consisting of cystic and solid components, usually with thick calcifications, and calcification occurs in 50% of cases. In addition, teratomas occur equally in females and males, but hairy polyps are more likely to affect females than males [14]. Teratomas can be malignant, but there have been no malignant reports on hairy polyps. Dermoids are cystic lesions containing desquamated epithelial products and keratinizing squamous epithelium [23], are more cystic than hairy polyps with a thin wall, and are usually found in the middle and front of the oral cavity [27]. The density or signal is usually nonuniform, with a fluid level and marble-like appearance by MRI and diffusion restriction [27]. The presence of soft tissue density or intensity in the middle ear cavity together with ossicular and mastoid bony erosion is highly specific for cholesteatoma. MR imaging reveals that cholesteatomas do not show any enhancement and demonstrate diffusion restriction on DWI, which can be differentiated from hairy polyps occurring in the middle ear whose soft tissue cores show slight enhancement, without restricted diffusion on DWI [28].

Considering that a large proportion of patients with hair polyps are associated with other congenital malformations that are mostly found within the imaging scope of their investigations, we recommend that all patients clinically suspected of this disease undergo CT and/or MRI examination. In general, it is not necessary to use contrast media for lesions with typical imaging features on non-contrast enhanced images. However, for lesions with atypical imaging findings or in rare locations such as the middle ear or parapharyngeal space, contrast enhanced scanning plays an important role in differential diagnosis and determination of the extent and blood supply of the lesion.

Surgical excision of the mass at the pedicle base is an effective way to treat hairy polyps [29]. Two cases of spontaneous autoamputation have also been described [30,31]. To date, there have been no reports about malignant transformation of these masses, and recurrence after surgical excision is extremely rare [32].

This study had several limitations. The first was the insufficient sample size for statistical measurements. Second, there was no consistent scanning protocol for each patient in this study. Third, there was no available genetic test result to provide evidence for the pathogenesis of associated malformations. Hence, further investigation with a larger cohort is required to understand the full disease spectrum among children with hairy polyps. Standard CT and MRI scanning procedures should be developed for head and neck masses, too, especially in those with fat. Future research should carry out genetic testing on patients with hairy polyps so as to have a more comprehensive understanding of the disease and its associated malformations.

## 5. Conclusions

Pharyngeal hairy polyps are sometimes associated with other congenital abnormalities that can be easily missed at an initial visit. Imaging is useful for delineating the size, origin, and extent of the mass, and it is essential for determining intracranial extension and evaluating associated comorbidities. There are some distinctive imaging features for pharyngeal hairy polyps, but sometimes their imaging findings can be atypical. For lesions with atypical imaging findings or in rare locations, we recommend more comprehensive imaging examinations, such as contrast enhanced CT and MRI.

## Figures and Tables

**Table 1:** Clinical and imaging features of patients with hairy polyps.

Case	Gender	Age at First Episode	Clinical Manifestation	Mass Origin	Mass Location	Radiological Examination	Shaped	Size (cm)	Imaging Features (Typical */Atypical)	Associated Abnormalities
1	Male	2 years and 8 months	Mouth breathing, hearing impairment, snoring	The back side of the left soft palate	Nasopharynx, oropharynx, laryngopharynx	CT	Tongue	2.7 × 1.7 × 1.1	Typical	# Ossicular chain disruption
2	Male	3 months	Nasal obstruction, polypnea	The right pharyngeal opening of auditory tube	Nasopharynx, oropharynx	CT	Tongue	4.1 × 1.8 × 0.9	Typical	
3	Male	1 day	Dyspnea, choking on milk	The back side of the left soft palate	Nasopharynx, oropharynx, laryngopharynx	CT	Tongue	1.1 × 1.1 × 2.5	Typical	
4	Female	1 day	Dyspnea, polypnea, perioralcyanosis, heart rate reduction, CPR (+)	The back side of the left soft palate	Nasopharynx, oropharynx	MRI, CT	Pear	2.3 × 1.4 × 1.1	Typical	A cystic lesion of liver
5	Female	1 day	Nasal obstruction	The right nasal vestibule	Nasal vestibule	MRI	Sausage	0.6 × 0.9 × 0.7	Atypical	# Frontal midline lipoma
6	Female	1 day	Dyspnea, polypnea, inspiratory stridor, choking on milk, CPR (+)	The soft palate	Nasal cavity, nasopharynx	MRI, CT, CEMRI, CECT	Irregular	1.7 × 1.5 × 3.7	Typical	# A cleft palate pectus excavatum
7	Female	29 days	Polypnea, gurgling with sputum, heart rate reduction, CPR (+)	The inferior side of the left pharyngeal opening of auditory tube	Nasopharynx, oropharynx, laryngopharynx	CT, CECT	Sausage	1.1 × 1.0 × 2.1	Typical	
8	Female	1 day	Difficulty feeding, nasal obstruction, mouth breathing	The back side of the left soft palate	Nasopharynx	MRI, CT	Irregular	1.5 × 1.1 × 1.2	Atypical	# A cleft palate, ameloblastic fibro-odontoma of mandible and malformation of sphenoid bone, highly suspicious ectopic teeth and the remnant of craniopharyngeal canal development
9	Female	1 day	Hearing impairment, otopyorrhea	The left parapharyngeal space	The parapharyngeal space, external auditory canal, and tympanum	MRI, CT, CEMRI	Irregular	2.5 × 3.6 × 4.3	Atypical	
10	Female	10 days	Dyspnea, perioralcyanosis	The left eustachian tube	Nasopharynx, oropharynx	CT	Sausage	2.8 × 0.9 × 0.9	Typical	
11	Female	4 days	Dyspnea, perioralcyanosis	The right pharyngeal recess	Nasopharynx, oropharynx, laryngopharynx	CT	Sausage	2.2 × 1.3 × 0.9	Typical	# First branchial fistula
12	Female	1 day	Mouth breathing, snoring	The back side of the left soft palate	Nasopharynx, oropharynx	MRI	Sausage	0.9 × 0.8 × 2.0	Typical	
13	Female	1 day	Dyspnea, polypnea, snoring, choking on milk	The back side of the right soft palate	Nasopharynx, oropharynx, laryngopharynx	MRI, CT	Pear	2.3 × 1.3 × 1.3	Typical	
14	Female	2 years and 5 months	Asymptomatic	The back side of the left soft palate	Nasopharynx, oropharynx, laryngopharynx	MRI	Tongue	2.5 × 1.5 × 1.0	Typical	

Typical *: Pedicled masses containing fat and a central core of soft tissue; #: Incidental findings within the imaging scope of their investigations.

## Data Availability

Not applicable.

## References

[1] Aughton D.J., Sloan C.T., Milad M.P., Huang T.E., Michael C., Harper C. (1990). Nasopharyngeal teratoma (‘hairy polyp’), Dandy-Walker malformation, diaphragmatic hernia, and other anomalies in a female infant. J. Med. Genet..

[2] Simmonds J.C., Jabbour J., Vaughn J.A., Paulson V.A., Poe D.S., Rahbar R. (2019). Hairy polyps: A new case presentation and a pathogenetic hypothesis. Laryngoscope.

[3] Chakravarti A., Vishwakarma S.K., Arora V.K., Singh I. (1998). Dermoid (hairy polyp) of the nasopharynx. Indian J. Pediatr..

[4] Çay A., Bektas D., Imamoglu M., Bahadir O., Cobanoglu U., Sarihan H. (2004). Oral teratoma: A case report and literature review. Pediatr. Surg. Int..

[5] Dutta M., Roy S., Ghatak S. (2015). Naso-oropharyngeal choristoma (hairy polyps): An overview and current update on presentation, management, origin and related controversies. Eur. Arch. Oto-Rhino-Laryngol..

[6] Kalcioglu M.T., Can S., Aydin N.E. (2010). Unusual case of soft palate hairy polyp causing airway obstruction and review of the literature. J. Pediatr. Surg..

[7] Gambino M., Cozzi D.A., Aceti MG R., Manfredi P., Riccipetitoni G. (2008). Two unusual cases of pharyngeal hairy polyp causing intermittent neonatal airway obstruction. Int. J. Oral Maxillofac. Surg..

[8] Budenz C.L., Lesperance M.M., Gebarski S. (2005). Hairy polyp of the pharynx obscured on physical examination by endotracheal tube, but diagnosed on brain imaging. Pediatr. Radiol..

[9] Koike Y., Uchida K., Inoue M., Ohtsu K., Tanaka T., Otake K., Tanaka K., Kusunoki M. (2013). Hairy polyp can be lethal even when small in size. Pediatr. Int. Off. J. Jpn. Pediatr. Soc..

[10] Kaplan Y., Ulkumen B., Gokpınar S., Senel Z. (2015). Adult nasopharyngeal hairy polyp presenting with middle ear effusion. Med. Sci. Discov..

[11] Green V.S., Pearl G.S. (2006). A 24-year-old woman with a nasopharyngeal mass. Benign nasopharyngeal hairy polyp. Arch. Pathol. Lab. Med..

[12] Planas S., Ferreres J.C., Ortega A., Higueras T., Carreras E., Ramón y Cajal S., Torán N. (2009). Association of congenital hypothalamic hamartoma and hairy polyp. Ultrasound Obstet. Gynecol. Off. J. Int. Soc. Ultrasound Obstet. Gynecol..

[13] Barnes L., Eveson J.W., Sidransky D., Reichart P. (2005). Pathology and Genetics of Head and Neck Tumours.

[14] Jarvis S.J., Bull P.D. (2002). Hairy polyps of the nasopharynx. J. Laryngol. Otol..

[15] Kown J.-H., Lee J.-H. (2020). Hairy Polyp Arising from the Nasal Vestibule. Ear Nose Throat J..

[16] Teng Y., Xian Z., Han S., Liang Z., Pan H., Li L. (2019). Pharyngeal hairy polyps: Case series and literature review. Medicine.

[17] Wu J., Schulte J., Yang C., Baroody F., Ginat D.T. (2016). Hairy Polyp of the Nasopharynx Arising from the Eustachian Tube. Head Neck Pathol..

[18] Kraft J.K., Knight L.C., Cullinane C. (2011). US and MRI of a pharyngeal hairy polyp with pathological correlation. Pediatr. Radiol..

[19] Yilmaz M., Ibrahimov M., Ozturk O., Karaman E., Aslan M. (2012). Congenital hairy polyp of the soft palate. Int. J. Pediatr. Otorhinolaryngol..

[20] Vaughan C., Prowse S.J., Knight L.C. (2012). Hairy polyp of the oropharynx in association with a first branchial arch sinus. J. Laryngol. Otol..

[21] Tariq M.U., Din N.U., Bashir M.R. (2013). Hairy polyp, a clinicopathologic study of four cases. Head Neck Pathol..

[22] Johnston D.R., Whittemore K., Poe D., Robson C.D., Perez-Atayde A.R. (2011). Diagnostic and surgical challenge: Middle ear dermoid cyst in 12 month old with branchio-oto-renal syndrome and multiple middle-ear congenital anomalies. Int. J. Pediatr. Otorhinolaryngol..

[23] Sheng M., Mi Y., Gao F., Liang J., Zhou H. (2021). Imaging features of pharyngeal hairy polyps in infants. Oral Radiol..

[24] Lansford C.D., Bossen E.H., Scher R.L. (2003). Pathology quiz case 1. Hairy polyp. Arch. Otolaryngol.—Head Neck Surg..

[25] Chang S.S., Halushka M., Vander Meer J., Goins M., Francis H.W. (2008). Nasopharyngeal hairy polyp with recurrence in the middle ear. Int. J. Pediatr. Otorhinolaryngol..

[26] Christianson B., Ulualp S.O., Koral K., Rakheja D., Deskin R. (2013). Congenital hairy polyp of the palatopharyngeus muscle. Case Rep. Otolaryngol..

[27] Edwards R.M., Chapman T., Horn D.L., Paladin A.M., Iyer R.S. (2013). Imaging of pediatric floor of mouth lesions. Pediatr. Radiol..

[28] Gulati M., Gupta S., Prakash A., Garg A., Dixit R. (2019). HRCT imaging of acquired cholesteatoma: A pictorial review. Insights Imaging.

[29] Roh J.-L. (2004). Transoral endoscopic resection of a nasopharyngeal hairy polyp. Int. J. Pediatr. Otorhinolaryngol..

[30] Algaberi A.K., Alhwish M.A., Alshoabi S.A., Alhazmi F.H., Alsultan K.D., Hamid A.M. (2021). Autoamputated pharyngeal hairy polyp presented with aero-digestive obstruction: A case report. Radiol. Case Rep..

[31] De Caluwe D., Kealey S., Hayes R., Puri P. (2002). Autoamputation of a congenital oropharyngeal hairy polyp. Pediatr. Surg. Int..

[32] Chaudhry A.P., Lore J.M., Fisher J.E., Gambrino A.G. (1978). So-called hairy polyps or teratoid tumors of the nasopharynx. Arch. Otolaryngol..

